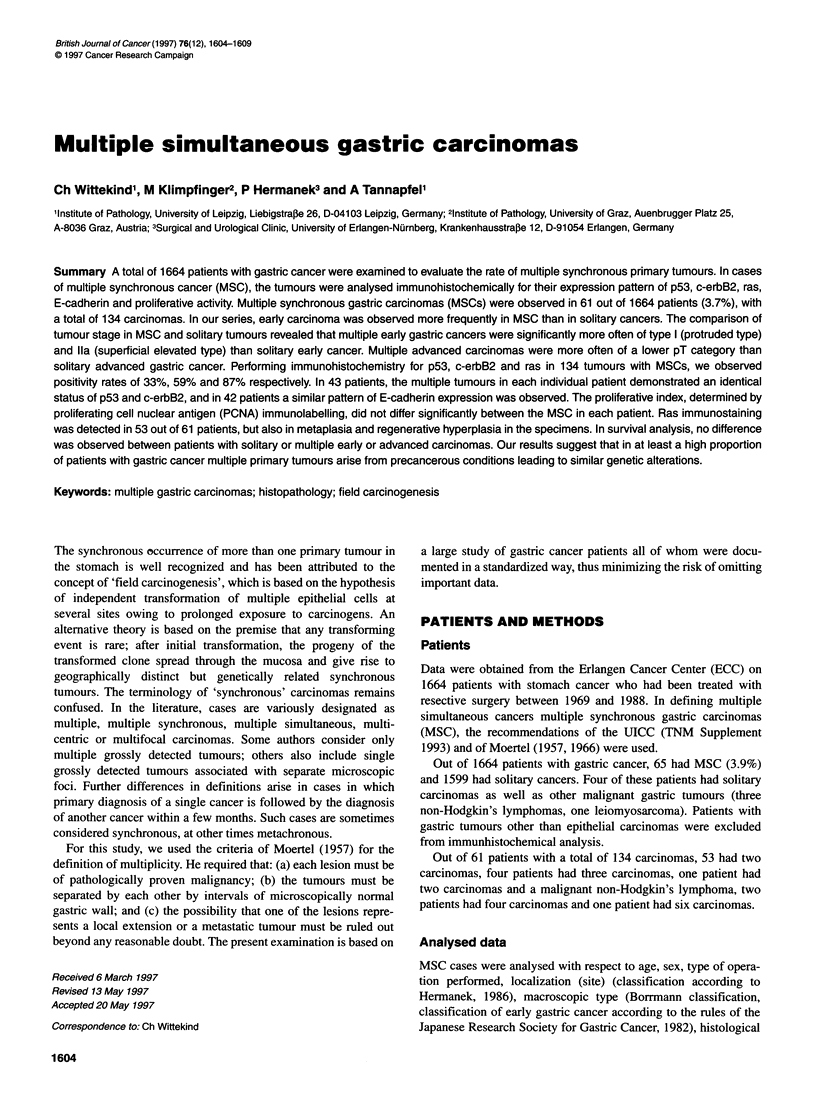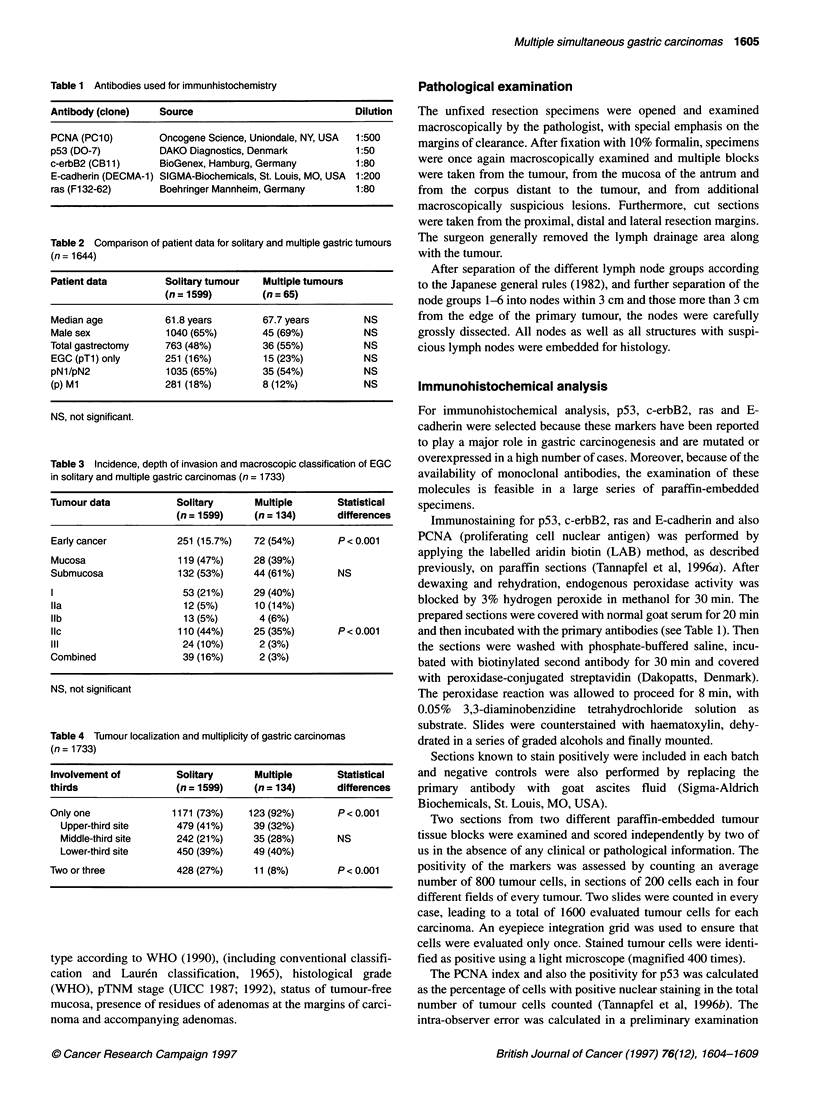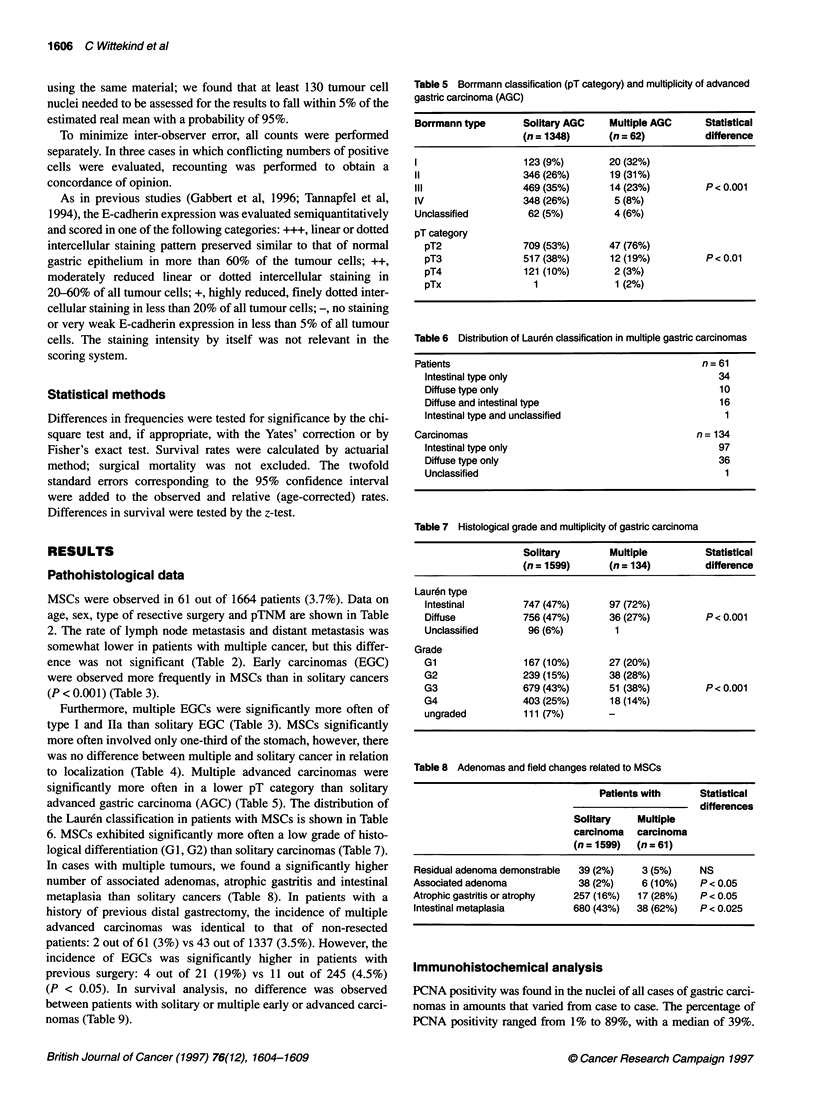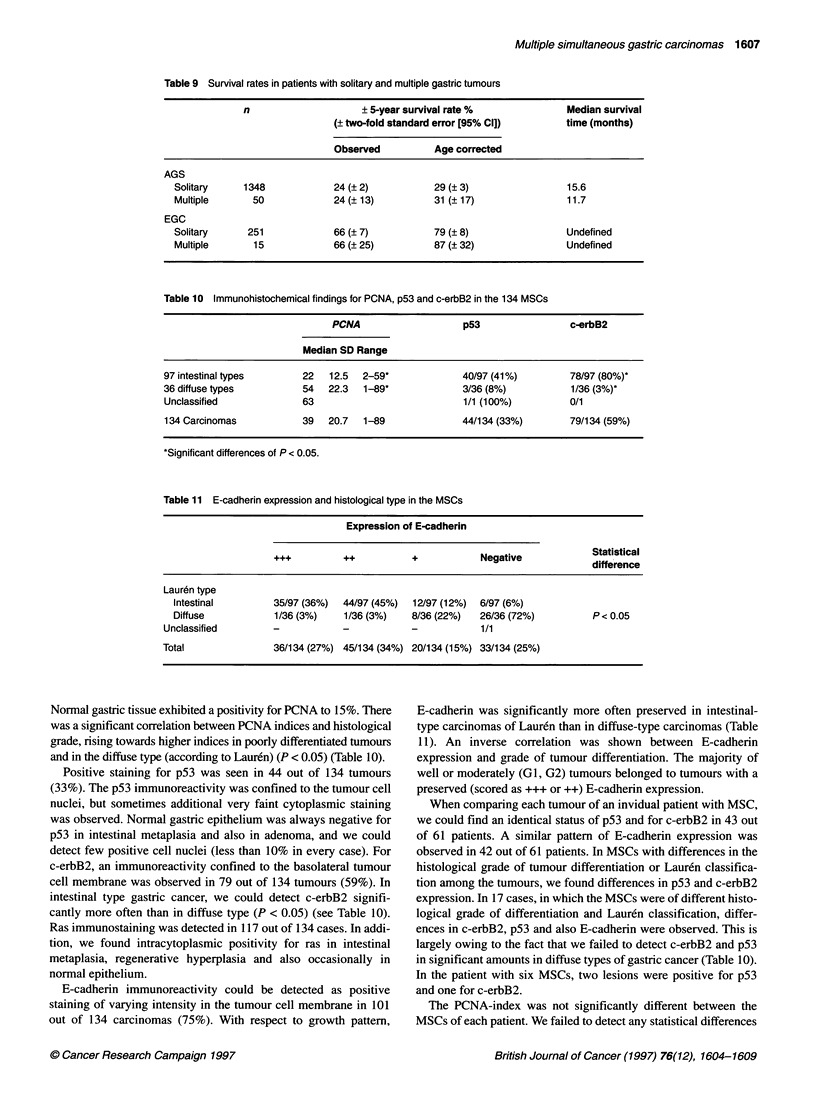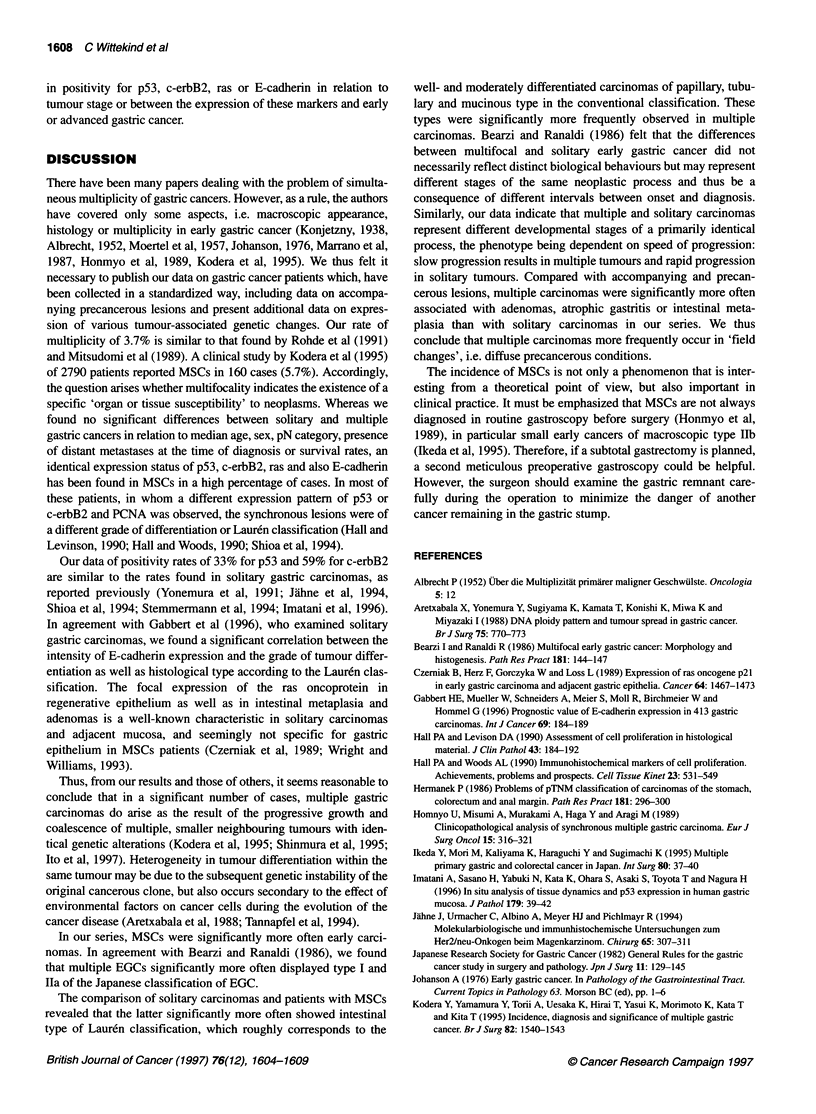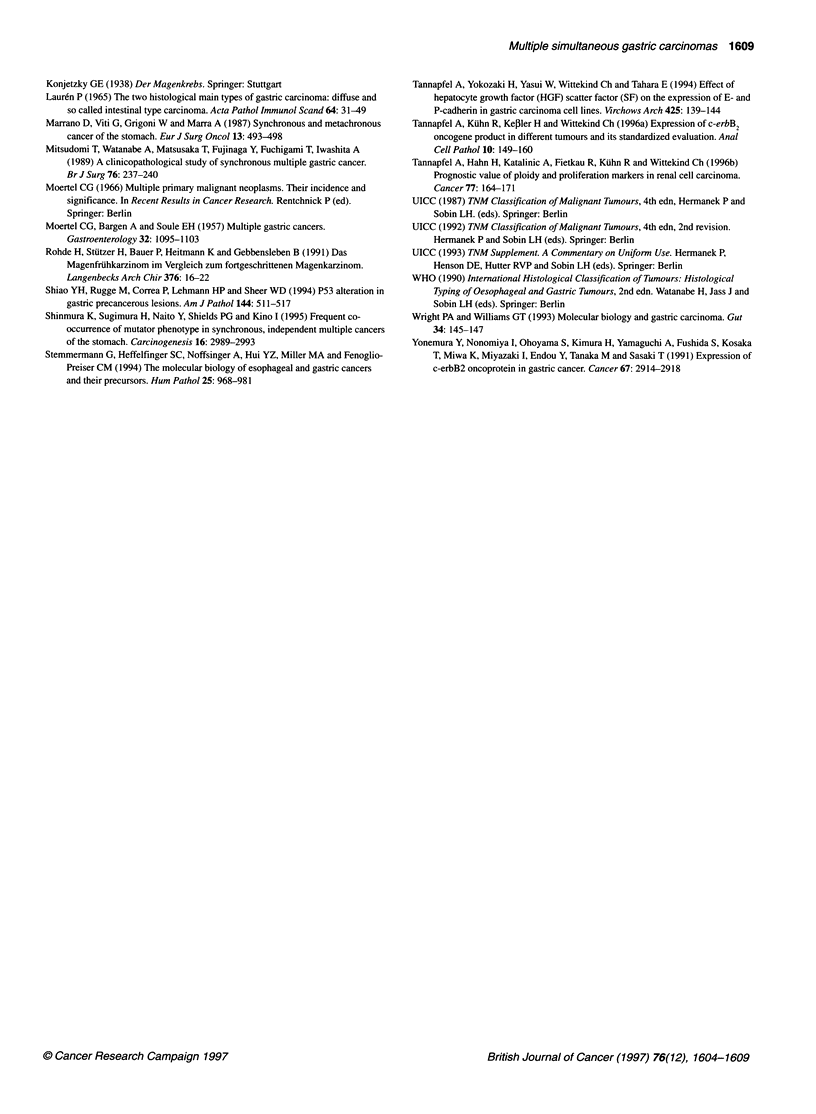# Multiple simultaneous gastric carcinomas.

**DOI:** 10.1038/bjc.1997.604

**Published:** 1997

**Authors:** C. Wittekind, M. Klimpfinger, P. Hermanek, A. Tannapfel

**Affiliations:** Institute of Pathology, University of Leipzig, Germany.

## Abstract

A total of 1664 patients with gastric cancer were examined to evaluate the rate of multiple synchronous primary tumours. In cases of multiple synchronous cancer (MSC), the tumours were analysed immunohistochemically for their expression pattern of p53, c-erbB2, ras, E-cadherin and proliferative activity. Multiple synchronous gastric carcinomas (MSCs) were observed in 61 out of 1664 patients (3.7%), with a total of 134 carcinomas. In our series, early carcinoma was observed more frequently in MSC than in solitary cancers. The comparison of tumour stage in MSC and solitary tumours revealed that multiple early gastric cancers were significantly more often of type I (protruded type) and IIa (superficial elevated type) than solitary early cancer. Multiple advanced carcinomas were more often of a lower pT category than solitary advanced gastric cancer. Performing immunohistochemistry for p53, c-erbB2 and ras in 134 tumours with MSCs, we observed positivity rates of 33%, 59% and 87% respectively. In 43 patients, the multiple tumours in each individual patient demonstrated an identical status of p53 and c-erbB2, and in 42 patients a similar pattern of E-cadherin expression was observed. The proliferative index, determined by proliferating cell nuclear antigen (PCNA) immunolabelling, did not differ significantly between the MSC in each patient. Ras immunostaining was detected in 53 out of 61 patients, but also in metaplasia and regenerative hyperplasia in the specimens. In survival analysis, no difference was observed between patients with solitary or multiple early or advanced carcinomas. Our results suggest that in at least a high proportion of patients with gastric cancer multiple primary tumours arise from precancerous conditions leading to similar genetic alterations.


British Journal of Cancer (1997) 76(12), 1604–1609
© 1997 Cancer Research Campaign

### Multiple simultaneous gastric carcinomas

**Ch Wittekind[1], M Klimpfinger[2], P Hermanek[3] and A Tannapfel[1]**

[1]Institute of Pathology, University of Leipzig, Liebigstraβe 26, D-04103 Leipzig, Germany; [2]Institute of Pathology, University of Graz, Auenbrugger Platz 25, A-8036 Graz, Austria; [3]Surgical and Urological Clinic, University of Erlangen-Nürnberg, Krankenhausstraβe 12, D-91054 Erlangen, Germany

**Summary** A total of 1664 patients with gastric cancer were examined to evaluate the rate of multiple synchronous primary tumours. In cases of multiple synchronous cancer (MSC), the tumours were analysed immunohistochemically for their expression pattern of p53, c-erbB2, ras, E-cadherin and proliferative activity. Multiple synchronous gastric carcinomas (MSCs) were observed in 61 out of 1664 patients (3.7%), with a total of 134 carcinomas. In our series, early carcinoma was observed more frequently in MSC than in solitary cancers. The comparison of tumour stage in MSC and solitary tumours revealed that multiple early gastric cancers were significantly more often of type I (protruded type) and IIa (superficial elevated type) than solitary early cancer. Multiple advanced carcinomas were more often of a lower pT category than solitary advanced gastric cancer. Performing immunohistochemistry for p53, c-erbB2 and ras in 134 tumours with MSCs, we observed positivity rates of 33%, 59% and 87% respectively. In 43 patients, the multiple tumours in each individual patient demonstrated an identical status of p53 and c-erbB2, and in 42 patients a similar pattern of E-cadherin expression was observed. The proliferative index, determined by proliferating cell nuclear antigen (PCNA) immunolabelling, did not differ significantly between the MSC in each patient. Ras immunostaining was detected in 53 out of 61 patients, but also in metaplasia and regenerative hyperplasia in the specimens. In survival analysis, no difference was observed between patients with solitary or multiple early or advanced carcinomas. Our results suggest that in at least a high proportion of patients with gastric cancer multiple primary tumours arise from precancerous conditions leading to similar genetic alterations.

**Keywords:** multiple gastric carcinomas; histopathology; field carcinogenesis

The synchronous occurrence of more than one primary tumour in the stomach is well recognized and has been attributed to the concept of 'field carcinogenesis', which is based on the hypothesis of independent transformation of multiple epithelial cells at several sites owing to prolonged exposure to carcinogens. An alternative theory is based on the premise that any transforming event is rare; after initial transformation, the progeny of the transformed clone spread through the mucosa and give rise to geographically distinct but genetically related synchronous tumours. The terminology of 'synchronous' carcinomas remains confused. In the literature, cases are variously designated as multiple, multiple synchronous, multiple simultaneous, multicentric or multifocal carcinomas. Some authors consider only multiple grossly detected tumours; others also include single grossly detected tumours associated with separate microscopic foci. Further differences in definitions arise in cases in which primary diagnosis of a single cancer is followed by the diagnosis of another cancer within a few months. Such cases are sometimes considered synchronous, at other times metachronous.

For this study, we used the criteria of Moertel (1957) for the definition of multiplicity. He required that: (a) each lesion must be of pathologically proven malignancy; (b) the tumours must be separated by each other by intervals of microscopically normal gastric wall; and (c) the possibility that one of the lesions represents a local extension or a metastatic tumour must be ruled out beyond any reasonable doubt. The present examination is based on a large study of gastric cancer patients all of whom were documented in a standardized way, thus minimizing the risk of omitting important data.

*Received 6 March 1997*
*Revised 13 May 1997*
*Accepted 20 May 1997*

*Correspondence to:* Ch Wittekind

### PATIENTS AND METHODS

### Patients

Data were obtained from the Erlangen Cancer Center (ECC) on 1664 patients with stomach cancer who had been treated with resective surgery between 1969 and 1988. In defining multiple simultaneous cancers multiple synchronous gastric carcinomas (MSC), the recommendations of the UICC (TNM Supplement 1993) and of Moertel (1957, 1966) were used.

Out of 1664 patients with gastric cancer, 65 had MSC (3.9%) and 1599 had solitary cancers. Four of these patients had solitary carcinomas as well as other malignant gastric tumours (three non-Hodgkin's lymphomas, one leiomyosarcoma). Patients with gastric tumours other than epithelial carcinomas were excluded from immunhistochemical analysis.

Out of 61 patients with a total of 134 carcinomas, 53 had two carcinomas, four patients had three carcinomas, one patient had two carcinomas and a malignant non-Hodgkin's lymphoma, two patients had four carcinomas and one patient had six carcinomas.

### Analysed data

MSC cases were analysed with respect to age, sex, type of operation performed, localization (site) (classification according to Hermanek, 1986), macroscopic type (Borrmann classification, classification of early gastric cancer according to the rules of the Japanese Research Society for Gastric Cancer, 1982), histological

**Table 1** Antibodies used for immunhistochemistry

| Antibody (clone) | Source | Dilution |
|---|---|---|
| PCNA (PC10) | Oncogene Science, Uniondale, NY, USA | 1:500 |
| p53 (DO-7) | DAKO Diagnostics, Denmark | 1:50 |
| c-erbB2 (CB11) | BioGenex, Hamburg, Germany | 1:80 |
| E-cadherin (DECMA-1) | SIGMA-Biochemicals, St. Louis, MO, USA | 1:200 |
| ras (F132-62) | Boehringer Mannheim, Germany | 1:80 |

**Table 2** Comparison of patient data for solitary and multiple gastric tumours ($n$ = 1644)

| Patient data | Solitary tumour ($n$ = 1599) | Multiple tumours ($n$ = 65) | |
|---|---|---|---|
| Median age | 61.8 years | 67.7 years | NS |
| Male sex | 1040 (65%) | 45 (69%) | NS |
| Total gastrectomy | 763 (48%) | 36 (55%) | NS |
| EGC (pT1) only | 251 (16%) | 15 (23%) | NS |
| pN1/pN2 | 1035 (65%) | 35 (54%) | NS |
| (p) M1 | 281 (18%) | 8 (12%) | NS |

NS, not significant.

**Table 3** Incidence, depth of invasion and macroscopic classification of EGC in solitary and multiple gastric carcinomas ($n$ = 1733)

| Tumour data | Solitary ($n$ = 1599) | Multiple ($n$ = 134) | Statistical differences |
|---|---|---|---|
| Early cancer | 251 (15.7%) | 72 (54%) | $P < 0.001$ |
| Mucosa | 119 (47%) | 28 (39%) | |
| Submucosa | 132 (53%) | 44 (61%) | NS |
| I | 53 (21%) | 29 (40%) | |
| IIa | 12 (5%) | 10 (14%) | |
| IIb | 13 (5%) | 4 (6%) | |
| IIc | 110 (44%) | 25 (35%) | $P < 0.001$ |
| III | 24 (10%) | 2 (3%) | |
| Combined | 39 (16%) | 2 (3%) | |

NS, not significant

**Table 4** Tumour localization and multiplicity of gastric carcinomas ($n$ = 1733)

| Involvement of thirds | Solitary ($n$ = 1599) | Multiple ($n$ = 134) | Statistical differences |
|---|---|---|---|
| Only one | 1171 (73%) | 123 (92%) | $P < 0.001$ |
| Upper-third site | 479 (41%) | 39 (32%) | |
| Middle-third site | 242 (21%) | 35 (28%) | NS |
| Lower-third site | 450 (39%) | 49 (40%) | |
| Two or three | 428 (27%) | 11 (8%) | $P < 0.001$ |

type according to WHO (1990), (including conventional classification and Laurén classification, 1965), histological grade (WHO), pTNM stage (UICC 1987; 1992), status of tumour-free mucosa, presence of residues of adenomas at the margins of carcinoma and accompanying adenomas.

### Pathological examination

The unfixed resection specimens were opened and examined macroscopically by the pathologist, with special emphasis on the margins of clearance. After fixation with 10% formalin, specimens were once again macroscopically examined and multiple blocks were taken from the tumour, from the mucosa of the antrum and from the corpus distant to the tumour, and from additional macroscopically suspicious lesions. Furthermore, cut sections were taken from the proximal, distal and lateral resection margins. The surgeon generally removed the lymph drainage area along with the tumour.

After separation of the different lymph node groups according to the Japanese general rules (1982), and further separation of the node groups 1–6 into nodes within 3 cm and those more than 3 cm from the edge of the primary tumour, the nodes were carefully grossly dissected. All nodes as well as all structures with suspicious lymph nodes were embedded for histology.

### Immunohistochemical analysis

For immunohistochemical analysis, p53, c-erbB2, ras and E-cadherin were selected because these markers have been reported to play a major role in gastric carcinogenesis and are mutated or overexpressed in a high number of cases. Moreover, because of the availability of monoclonal antibodies, the examination of these molecules is feasible in a large series of paraffin-embedded specimens.

Immunostaining for p53, c-erbB2, ras and E-cadherin and also PCNA (proliferating cell nuclear antigen) was performed by applying the labelled aridin biotin (LAB) method, as described previously, on paraffin sections (Tannapfel et al, 1996*a*). After dewaxing and rehydration, endogenous peroxidase activity was blocked by 3% hydrogen peroxide in methanol for 30 min. The prepared sections were covered with normal goat serum for 20 min and then incubated with the primary antibodies (see Table 1). Then the sections were washed with phosphate-buffered saline, incubated with biotinylated second antibody for 30 min and covered with peroxidase-conjugated streptavidin (Dakopatts, Denmark). The peroxidase reaction was allowed to proceed for 8 min, with 0.05% 3,3-diaminobenzidine tetrahydrochloride solution as substrate. Slides were counterstained with haematoxylin, dehydrated in a series of graded alcohols and finally mounted.

Sections known to stain positively were included in each batch and negative controls were also performed by replacing the primary antibody with goat ascites fluid (Sigma-Aldrich Biochemicals, St. Louis, MO, USA).

Two sections from two different paraffin-embedded tumour tissue blocks were examined and scored independently by two of us in the absence of any clinical or pathological information. The positivity of the markers was assessed by counting an average number of 800 tumour cells, in sections of 200 cells each in four different fields of every tumour. Two slides were counted in every case, leading to a total of 1600 evaluated tumour cells for each carcinoma. An eyepiece integration grid was used to ensure that cells were evaluated only once. Stained tumour cells were identified as positive using a light microscope (magnified 400 times).

The PCNA index and also the positivity for p53 was calculated as the percentage of cells with positive nuclear staining in the total number of tumour cells counted (Tannapfel et al, 1996*b*). The intra-observer error was calculated in a preliminary examination using the same material; we found that at least 130 tumour cell nuclei needed to be assessed for the results to fall within 5% of the estimated real mean with a probability of 95%.

To minimize inter-observer error, all counts were performed separately. In three cases in which conflicting numbers of positive cells were evaluated, recounting was performed to obtain a concordance of opinion.

As in previous studies (Gabbert et al, 1996; Tannapfel et al, 1994), the E-cadherin expression was evaluated semiquantitatively and scored in one of the following categories: +++, linear or dotted intercellular staining pattern preserved similar to that of normal gastric epithelium in more than 60% of the tumour cells; ++, moderately reduced linear or dotted intercellular staining in 20–60% of all tumour cells; +, highly reduced, finely dotted intercellular staining in less than 20% of all tumour cells; –, no staining or very weak E-cadherin expression in less than 5% of all tumour cells. The staining intensity by itself was not relevant in the scoring system.

### Statistical methods

Differences in frequencies were tested for significance by the chi-square test and, if appropriate, with the Yates' correction or by Fisher's exact test. Survival rates were calculated by actuarial method; surgical mortality was not excluded. The twofold standard errors corresponding to the 95% confidence interval were added to the observed and relative (age-corrected) rates. Differences in survival were tested by the $z$-test.

### RESULTS

### Pathohistological data

MSCs were observed in 61 out of 1664 patients (3.7%). Data on age, sex, type of resective surgery and pTNM are shown in Table 2. The rate of lymph node metastasis and distant metastasis was somewhat lower in patients with multiple cancer, but this difference was not significant (Table 2). Early carcinomas (EGC) were observed more frequently in MSCs than in solitary cancers ($P < 0.001$) (Table 3).

Furthermore, multiple EGCs were significantly more often of type I and IIa than solitary EGC (Table 3). MSCs significantly more often involved only one-third of the stomach, however, there was no difference between multiple and solitary cancer in relation to localization (Table 4). Multiple advanced carcinomas were significantly more often in a lower pT category than solitary advanced gastric carcinoma (AGC) (Table 5). The distribution of the Laurén classification in patients with MSCs is shown in Table 6. MSCs exhibited significantly more often a low grade of histological differentiation (G1, G2) than solitary carcinomas (Table 7). In cases with multiple tumours, we found a significantly higher number of associated adenomas, atrophic gastritis and intestinal metaplasia than solitary cancers (Table 8). In patients with a history of previous distal gastrectomy, the incidence of multiple advanced carcinomas was identical to that of non-resected patients: 2 out of 61 (3%) vs 43 out of 1337 (3.5%). However, the incidence of EGCs was significantly higher in patients with previous surgery: 4 out of 21 (19%) vs 11 out of 245 (4.5%) ($P < 0.05$). In survival analysis, no difference was observed between patients with solitary or multiple early or advanced carcinomas (Table 9).

**Table 5** Borrmann classification (pT category) and multiplicity of advanced gastric carcinoma (AGC)

| Borrmann type | Solitary AGC ($n$ = 1348) | Multiple AGC ($n$ = 62) | Statistical difference |
|---|---|---|---|
| I | 123 (9%) | 20 (32%) | |
| II | 346 (26%) | 19 (31%) | |
| III | 469 (35%) | 14 (23%) | $P < 0.001$ |
| IV | 348 (26%) | 5 (8%) | |
| Unclassified | 62 (5%) | 4 (6%) | |
| pT category | | | |
| pT2 | 709 (53%) | 47 (76%) | |
| pT3 | 517 (38%) | 12 (19%) | $P < 0.01$ |
| pT4 | 121 (10%) | 2 (3%) | |
| pTx | 1 | 1 (2%) | |

**Table 6** Distribution of Laurén classification in multiple gastric carcinomas

| | |
|---|---|
| Patients | $n$ = 61 |
| Intestinal type only | 34 |
| Diffuse type only | 10 |
| Diffuse and intestinal type | 16 |
| Intestinal type and unclassified | 1 |
| Carcinomas | $n$ = 134 |
| Intestinal type only | 97 |
| Diffuse type only | 36 |
| Unclassified | 1 |

**Table 7** Histological grade and multiplicity of gastric carcinoma

| | Solitary ($n$ = 1599) | Multiple ($n$ = 134) | Statistical difference |
|---|---|---|---|
| Laurén type | | | |
| Intestinal | 747 (47%) | 97 (72%) | |
| Diffuse | 756 (47%) | 36 (27%) | $P < 0.001$ |
| Unclassified | 96 (6%) | 1 | |
| Grade | | | |
| G1 | 167 (10%) | 27 (20%) | |
| G2 | 239 (15%) | 38 (28%) | |
| G3 | 679 (43%) | 51 (38%) | $P < 0.001$ |
| G4 | 403 (25%) | 18 (14%) | |
| ungraded | 111 (7%) | – | |

**Table 8** Adenomas and field changes related to MSCs

| | Patients with | | Statistical differences |
|---|---|---|---|
| | Solitary carcinoma ($n$ = 1599) | Multiple carcinoma ($n$ = 61) | |
| Residual adenoma demonstrable | 39 (2%) | 3 (5%) | NS |
| Associated adenoma | 38 (2%) | 6 (10%) | $P < 0.05$ |
| Atrophic gastritis or atrophy | 257 (16%) | 17 (28%) | $P < 0.05$ |
| Intestinal metaplasia | 680 (43%) | 38 (62%) | $P < 0.025$ |

### Immunohistochemical analysis

PCNA positivity was found in the nuclei of all cases of gastric carcinomas in amounts that varied from case to case. The percentage of PCNA positivity ranged from 1% to 89%, with a median of 39%.

© Cancer Research Campaign 1997

**Table 9** Survival rates in patients with solitary and multiple gastric tumours

| | *n* | ± 5-year survival rate % (± two-fold standard error [95% CI]) | | Median survival time (months) |
|---|---|---|---|---|
| | | Observed | Age corrected | |
| AGS | | | | |
| Solitary | 1348 | 24 (± 2) | 29 (± 3) | 15.6 |
| Multiple | 50 | 24 (± 13) | 31 (± 17) | 11.7 |
| EGC | | | | |
| Solitary | 251 | 66 (± 7) | 79 (± 8) | Undefined |
| Multiple | 15 | 66 (± 25) | 87 (± 32) | Undefined |

**Table 10** Immunohistochemical findings for PCNA, p53 and c-erbB2 in the 134 MSCs

| | *PCNA* | | | p53 | c-erbB2 |
|---|---|---|---|---|---|
| | Median | SD | Range | | |
| 97 intestinal types | 22 | 12.5 | 2–59* | 40/97 (41%) | 78/97 (80%)* |
| 36 diffuse types | 54 | 22.3 | 1–89* | 3/36 (8%) | 1/36 (3%)* |
| Unclassified | 63 | | | 1/1 (100%) | 0/1 |
| 134 Carcinomas | 39 | 20.7 | 1–89 | 44/134 (33%) | 79/134 (59%) |

*Significant differences of $P < 0.05$.

**Table 11** E-cadherin expression and histological type in the MSCs

| | Expression of E-cadherin | | | | Statistical difference |
|---|---|---|---|---|---|
| | +++ | ++ | + | Negative | |
| Laurén type | | | | | |
| Intestinal | 35/97 (36%) | 44/97 (45%) | 12/97 (12%) | 6/97 (6%) | |
| Diffuse | 1/36 (3%) | 1/36 (3%) | 8/36 (22%) | 26/36 (72%) | $P < 0.05$ |
| Unclassified | – | – | – | 1/1 | |
| Total | 36/134 (27%) | 45/134 (34%) | 20/134 (15%) | 33/134 (25%) | |

Normal gastric tissue exhibited a positivity for PCNA to 15%. There was a significant correlation between PCNA indices and histological grade, rising towards higher indices in poorly differentiated tumours and in the diffuse type (according to Laurén) ($P < 0.05$) (Table 10).

Positive staining for p53 was seen in 44 out of 134 tumours (33%). The p53 immunoreactivity was confined to the tumour cell nuclei, but sometimes additional very faint cytoplasmic staining was observed. Normal gastric epithelium was always negative for p53 in intestinal metaplasia and also in adenoma, and we could detect few positive cell nuclei (less than 10% in every case). For c-erbB2, an immunoreactivity confined to the basolateral tumour cell membrane was observed in 79 out of 134 tumours (59%). In intestinal type gastric cancer, we could detect c-erbB2 significantly more often than in diffuse type ($P < 0.05$) (see Table 10). Ras immunostaining was detected in 117 out of 134 cases. In addition, we found intracytoplasmic positivity for ras in intestinal metaplasia, regenerative hyperplasia and also occasionally in normal epithelium.

E-cadherin immunoreactivity could be detected as positive staining of varying intensity in the tumour cell membrane in 101 out of 134 carcinomas (75%). With respect to growth pattern, E-cadherin was significantly more often preserved in intestinal-type carcinomas of Laurén than in diffuse-type carcinomas (Table 11). An inverse correlation was shown between E-cadherin expression and grade of tumour differentiation. The majority of well or moderately (G1, G2) tumours belonged to tumours with a preserved (scored as +++ or ++) E-cadherin expression.

When comparing each tumour of an invidual patient with MSC, we could find an identical status of p53 and for c-erbB2 in 43 out of 61 patients. A similar pattern of E-cadherin expression was observed in 42 out of 61 patients. In MSCs with differences in the histological grade of tumour differentiation or Laurén classification among the tumours, we found differences in p53 and c-erbB2 expression. In 17 cases, in which the MSCs were of different histological grade of differentiation and Laurén classification, differences in c-erbB2, p53 and also E-cadherin were observed. This is largely owing to the fact that we failed to detect c-erbB2 and p53 in significant amounts in diffuse types of gastric cancer (Table 10). In the patient with six MSCs, two lesions were positive for p53 and one for c-erbB2.

The PCNA-index was not significantly different between the MSCs of each patient. We failed to detect any statistical differences

© Cancer Research Campaign 1997 

in positivity for p53, c-erbB2, ras or E-cadherin in relation to tumour stage or between the expression of these markers and early or advanced gastric cancer.

### DISCUSSION

There have been many papers dealing with the problem of simultaneous multiplicity of gastric cancers. However, as a rule, the authors have covered only some aspects, i.e. macroscopic appearance, histology or multiplicity in early gastric cancer (Konjetzny, 1938, Albrecht, 1952, Moertel et al, 1957, Johanson, 1976, Marrano et al, 1987, Honmyo et al, 1989, Kodera et al, 1995). We thus felt it necessary to publish our data on gastric cancer patients which, have been collected in a standardized way, including data on accompanying precancerous lesions and present additional data on expression of various tumour-associated genetic changes. Our rate of multiplicity of 3.7% is similar to that found by Rohde et al (1991) and Mitsudomi et al (1989). A clinical study by Kodera et al (1995) of 2790 patients reported MSCs in 160 cases (5.7%). Accordingly, the question arises whether multifocality indicates the existence of a specific 'organ or tissue susceptibility' to neoplasms. Whereas we found no significant differences between solitary and multiple gastric cancers in relation to median age, sex, pN category, presence of distant metastases at the time of diagnosis or survival rates, an identical expression status of p53, c-erbB2, ras and also E-cadherin has been found in MSCs in a high percentage of cases. In most of these patients, in whom a different expression pattern of p53 or c-erbB2 and PCNA was observed, the synchronous lesions were of a different grade of differentiation or Laurén classification (Hall and Levinson, 1990; Hall and Woods, 1990; Shioa et al, 1994).

Our data of positivity rates of 33% for p53 and 59% for c-erbB2 are similar to the rates found in solitary gastric carcinomas, as reported previously (Yonemura et al, 1991; Jähne et al, 1994, Shioa et al, 1994; Stemmermann et al, 1994; Imatani et al, 1996). In agreement with Gabbert et al (1996), who examined solitary gastric carcinomas, we found a significant correlation between the intensity of E-cadherin expression and the grade of tumour differentiation as well as histological type according to the Laurén classification. The focal expression of the ras oncoprotein in regenerative epithelium as well as in intestinal metaplasia and adenomas is a well-known characteristic in solitary carcinomas and adjacent mucosa, and seemingly not specific for gastric epithelium in MSCs patients (Czerniak et al, 1989; Wright and Williams, 1993).

Thus, from our results and those of others, it seems reasonable to conclude that in a significant number of cases, multiple gastric carcinomas do arise as the result of the progressive growth and coalescence of multiple, smaller neighbouring tumours with identical genetic alterations (Kodera et al, 1995; Shinmura et al, 1995; Ito et al, 1997). Heterogeneity in tumour differentiation within the same tumour may be due to the subsequent genetic instability of the original cancerous clone, but also occurs secondary to the effect of environmental factors on cancer cells during the evolution of the cancer disease (Aretxabala et al, 1988; Tannapfel et al, 1994).

In our series, MSCs were significantly more often early carcinomas. In agreement with Bearzi and Ranaldi (1986), we found that multiple EGCs significantly more often displayed type I and IIa of the Japanese classification of EGC.

The comparison of solitary carcinomas and patients with MSCs revealed that the latter significantly more often showed intestinal type of Laurén classification, which roughly corresponds to the well- and moderately differentiated carcinomas of papillary, tubulary and mucinous type in the conventional classification. These types were significantly more frequently observed in multiple carcinomas. Bearzi and Ranaldi (1986) felt that the differences between multifocal and solitary early gastric cancer did not necessarily reflect distinct biological behaviours but may represent different stages of the same neoplastic process and thus be a consequence of different intervals between onset and diagnosis. Similarly, our data indicate that multiple and solitary carcinomas represent different developmental stages of a primarily identical process, the phenotype being dependent on speed of progression: slow progression results in multiple tumours and rapid progression in solitary tumours. Compared with accompanying and precancerous lesions, multiple carcinomas were significantly more often associated with adenomas, atrophic gastritis or intestinal metaplasia than with solitary carcinomas in our series. We thus conclude that multiple carcinomas more frequently occur in 'field changes', i.e. diffuse precancerous conditions.

The incidence of MSCs is not only a phenomenon that is interesting from a theoretical point of view, but also important in clinical practice. It must be emphasized that MSCs are not always diagnosed in routine gastroscopy before surgery (Honmyo et al, 1989), in particular small early cancers of macroscopic type IIb (Ikeda et al, 1995). Therefore, if a subtotal gastrectomy is planned, a second meticulous preoperative gastroscopy could be helpful. However, the surgeon should examine the gastric remnant carefully during the operation to minimize the danger of another cancer remaining in the gastric stump.

### REFERENCES

Albrecht P (1952) Über die Multiplizität primärer maligner Geschwülste. *Oncologia* **5**: 12

Aretxabala X, Yonemura Y, Sugiyama K, Kamata T, Konishi K, Miwa K and Miyazaki I (1988) DNA ploidy pattern and tumour spread in gastric cancer. *Br J Surg* **75**: 770–773

Bearzi I and Ranaldi R (1986) Multifocal early gastric cancer: Morphology and histogenesis. *Path Res Pract* **181**: 144–147

Czerniak B, Herz F, Gorczyka W and Loss L (1989) Expression of ras oncogene p21 in early gastric carcinoma and adjacent gastric epithelia. *Cancer* **64**: 1467–1473

Gabbert HE, Mueller W, Schneiders A, Meier S, Moll R, Birchmeier W and Hommel G (1996) Prognostic value of E-cadherin expression in 413 gastric carcinomas. *Int J Cancer* **69**: 184–189

Hall PA and Levison DA (1990) Assessment of cell proliferation in histological material. *J Clin Pathol* **43**: 184–192

Hall PA and Woods AL (1990) Immunohistochemical markers of cell proliferation. Achievements, problems and prospects. *Cell Tissue Kinet* **23**: 531–549

Hermanek P (1986) Problems of pTNM classification of carcinomas of the stomach, colorectum and anal margin. *Path Res Pract* **181**: 296–300

Homnyo U, Misumi A, Murakami A, Haga Y and Aragi M (1989) Clinicopathological analysis of synchronous multiple gastric carcinoma. *Eur J Surg Oncol* **15**: 316–321

Ikeda Y, Mori M, Kaliyama K, Haraguchi Y and Sugimachi K (1995) Multiple primary gastric and colorectal cancer in Japan. *Int Surg* **80**: 37–40

Imatani A, Sasano H, Yabuki N, Kata K, Ohara S, Asaki S, Toyota T and Nagura H (1996) In situ analysis of tissue dynamics and p53 expression in human gastric mucosa. *J Pathol* **179**: 39–42

Jähne J, Urmacher C, Albino A, Meyer HJ and Pichlmayr R (1994) Molekularbiologische und immunhistochemische Untersuchungen zum Her2/neu-Onkogen beim Magenkarzinom. *Chirurg* **65**: 307–311

Japanese Research Society for Gastric Cancer (1982) General Rules for the gastric cancer study in surgery and pathology. *Jpn J Surg* **11**: 129–145

Johanson A (1976) Early gastric cancer. In *Pathology of the Gastrointestinal Tract. Current Topics in Pathology 63.* Morson BC (ed), pp. 1–6

Kodera Y, Yamamura Y, Torii A, Uesaka K, Hirai T, Yasui K, Morimoto K, Kata T and Kita T (1995) Incidence, diagnosis and significance of multiple gastric cancer. *Br J Surg* **82**: 1540–1543

© Cancer Research Campaign 1997

Konjetzky GE (1938) *Der Magenkrebs*. Springer: Stuttgart

Laurén P (1965) The two histological main types of gastric carcinoma: diffuse and so called intestinal type carcinoma. *Acta Pathol Immunol Scand* **64**: 31–49

Marrano D, Viti G, Grigoni W and Marra A (1987) Synchronous and metachronous cancer of the stomach. *Eur J Surg Oncol* **13**: 493–498

Mitsudomi T, Watanabe A, Matsusaka T, Fujinaga Y, Fuchigami T, Iwashita A (1989) A clinicopathological study of synchronous multiple gastric cancer. *Br J Surg* **76**: 237–240

Moertel CG (1966) Multiple primary malignant neoplasms. Their incidence and significance. In *Recent Results in Cancer Research*. Rentchnick P (ed). Springer: Berlin

Moertel CG, Bargen A and Soule EH (1957) Multiple gastric cancers. *Gastroenterology* **32**: 1095–1103

Rohde H, Stützer H, Bauer P, Heitmann K and Gebbensleben B (1991) Das Magenfrühkarzinom im Vergleich zum fortgeschrittenen Magenkarzinom. *Langenbecks Arch Chir* **376**: 16–22

Shiao YH, Rugge M, Correa P, Lehmann HP and Sheer WD (1994) P53 alteration in gastric precancerous lesions. *Am J Pathol* **144**: 511–517

Shinmura K, Sugimura H, Naito Y, Shields PG and Kino I (1995) Frequent co-occurrence of mutator phenotype in synchronous, independent multiple cancers of the stomach. *Carcinogenesis* **16**: 2989–2993

Stemmermann G, Heffelfinger SC, Noffsinger A, Hui YZ, Miller MA and Fenoglio-Preiser CM (1994) The molecular biology of esophageal and gastric cancers and their precursors. *Hum Pathol* **25**: 968–981

Tannapfel A, Yokozaki H, Yasui W, Wittekind Ch and Tahara E (1994) Effect of hepatocyte growth factor (HGF) scatter factor (SF) on the expression of E- and P-cadherin in gastric carcinoma cell lines. *Virchows Arch* **425**: 139–144

Tannapfel A, Kühn R, Keβler H and Wittekind Ch (1996a) Expression of c-*erb*$B_2$ oncogene product in different tumours and its standardized evaluation. *Anal Cell Pathol* **10**: 149–160

Tannapfel A, Hahn H, Katalinic A, Fietkau R, Kühn R and Wittekind Ch (1996b) Prognostic value of ploidy and proliferation markers in renal cell carcinoma. *Cancer* **77**: 164–171

UICC (1987) *TNM Classification of Malignant Tumours*, 4th edn, Hermanek P and Sobin LH. (eds). Springer: Berlin

UICC (1992) *TNM Classification of Malignant Tumours*, 4th edn, 2nd revision. Hermanek P and Sobin LH (eds). Springer: Berlin

UICC (1993) *TNM Supplement. A Commentary on Uniform Use.* Hermanek P, Henson DE, Hutter RVP and Sobin LH (eds). Springer: Berlin

WHO (1990) *International Histological Classification of Tumours: Histological Typing of Oesophageal and Gastric Tumours*, 2nd edn. Watanabe H, Jass J and Sobin LH (eds). Springer: Berlin

Wright PA and Williams GT (1993) Molecular biology and gastric carcinoma. *Gut* **34**: 145–147

Yonemura Y, Nonomiya I, Ohoyama S, Kimura H, Yamaguchi A, Fushida S, Kosaka T, Miwa K, Miyazaki I, Endou Y, Tanaka M and Sasaki T (1991) Expression of c-erbB2 oncoprotein in gastric cancer. *Cancer* **67**: 2914–2918

© Cancer Research Campaign 1997